# Effectiveness of atopic dermatitis patient education programs – a systematic review and meta-analysis

**DOI:** 10.1007/s00403-024-02871-y

**Published:** 2024-04-25

**Authors:** Luis F. Andrade, Parsa Abdi, Kayla D. Mashoudy, Amritpal Kooner, Ashley Egler, Rebecca Urbonas, Aaron Smith, Gil Yosipovitch

**Affiliations:** 1 Dr. Phillip Frost Department of Dermatology and Cutaneous Surgery, University of Miami Miller School of Medicine, 1150 NW 14th Street, Miami, FL 33136 USA; 2 Faculty of Medicine, Memorial University of Newfoundland, 300 Prince Philip Dr, St. John’s, NL A1B 3V6 Canada; 3 Chicago College of Osteopathic Medicine, Midwestern University, Downers Grove, IL 60515 USA; 4 Indiana University School of Medicine, 340 W. 10th Street, Fairbanks Hall 6217, Indianapolis, IN 46202 USA; 5 Charles E. Schmidt College of Medicine, Florida Atlantic University, 777 Glades Road BC-71, Boca Raton, FL 33431 USA; 6 University of Virginia School of Medicine, 1340 Jefferson Park Ave, Charlottesville, VA 22903 USA

**Keywords:** Atopic dermatitis, Eczema, Education, Public health, Dermatitis

## Abstract

Patient education in atopic dermatitis (AD) has worked in parallel to the gold standard of pharmacological treatment as a foundational component of therapeutic regimens. In addition to improving patient education, past investigations of educational interventions have demonstrated profound reductions in disease severity for patients living with AD. However, prior meta-analytical work has focused mostly on comparing in-person interventions, and thus the need to determine the effectiveness of virtual methodologies in the current post-COVID era remains. In this study, we conducted a systematic review of the literature to determine the effectiveness of online programming in AD education compared to in-person interventions. A comprehensive search was conducted in accordance with the Cochrane Handbook for Systematic Reviews of Interventions 2019. Studies were retrieved based on articles published up to 04 April 2023. Adherence to the Preferred Reporting Items for Systematic Review and Meta-Analysis (PRISMA) Statement guided the reportage process for this systematic review and meta-analysis. The primary outcome of our meta-analysis was the effect of various educational modalities on atopic dermatitis severity as measured by multiple scales across the studies, the most common including SCORAD, Dermatology Life Quality Index (DLQI), Patient Oriented Eczema Measure (POEM), and Eczema Area and Severity Index (EASI). Most studies were randomized controlled trials, primarily from North America and Western Europe and focused on patient and/or caregiver education about disease management, self-care techniques, avoidance of triggers, and comprehensive understanding of the disease process. Our pooled analyses showed that targeted educational programs in understudied adult populations can be as impactful as those in pediatric groups. Moreover, virtual interventions can be employed as constructive tools for reducing barriers of access to patient education. Future research on educational interventions should utilize various methodologies to encourage individual learning preferences with a focus on adult cohorts.

## Introduction

Atopic dermatitis (AD) is a chronic inflammatory skin disease with a very pronounced and detrimental impact on a patient’s quality of life. While the combination of topical and systematic treatments (e.g. corticosteroids, JAK-inhibitors, biologics) dependent on disease severity has been the gold standard of pharmacological interventions, patient education has worked in parallel as a foundational component of treatment regimens.

One of the earliest examples of successful educational programming can be seen in Germany, with the creation of ‘eczema academies’ in the 1990s deploying structured interventional sessions with modules based on general information about AD, practical advice for treatment of affected skin, education on triggers for flares, and discussion of the role of mental health in the disease. In the United States, the first eczema school was founded in 2014 with similar success at incorporating patient education to reduce disease severity and symptoms [1]. In addition to increases in patient education, prior studies of educational interventions have demonstrated a direct impact in reducing disease severity for pediatric patients living with AD [2].

However, prior meta-analytical work has focused mostly on comparing in-person interventions. Considering the large rise in virtual telehealth in the post-COVID era, we conducted a systematic review of the literature to determine the effectiveness of online programming in AD education compared to in-person interventions.

## Methodology

### Data source

A comprehensive search was conducted in accordance with the Cochrane Handbook for Systematic Reviews of Interventions 2019 [3]. The following databases were searched for articles on April 04, 2023, with no restriction imposed on publication date: PubMed, MEDLINE, CINAHL, Web of Sciences, Ovid Databases (e.g. Medline, Embase, Emcare). The inclusion and exclusion criteria were organized according to Population, Intervention, Comparison, Outcome, and Study (PICOS) criteria. Inclusion criteria: any age or gender patient with a diagnosis of atopic dermatitis (AD) and/or any parent/caregiver of a patient with a diagnosis of atopic dermatitis; any intervention with an educational focus aimed at improving health knowledge and decreasing severity of AD; with or without a comparator; primary outcomes include outcomes such as SCORing Atopic Dermatitis (SCORAD). Study design is limited to interventional studies in humans, published in English language in peer-reviewed journals. Exclusion criteria: no report of any primary outcome with raw data; secondary research (review or meta-analysis); gray literature (dissertation, conference abstract, letter, review, commentary, editorial, note); language other than English. No language or date restrictions were applied.

### Study selection

Studies were retrieved based on articles published up to 04 April 2023 with the intention of selecting for articles incorporating educational interventions for patients and/or caregivers of patients living with atopic dermatitis.

**Fig. 1:**
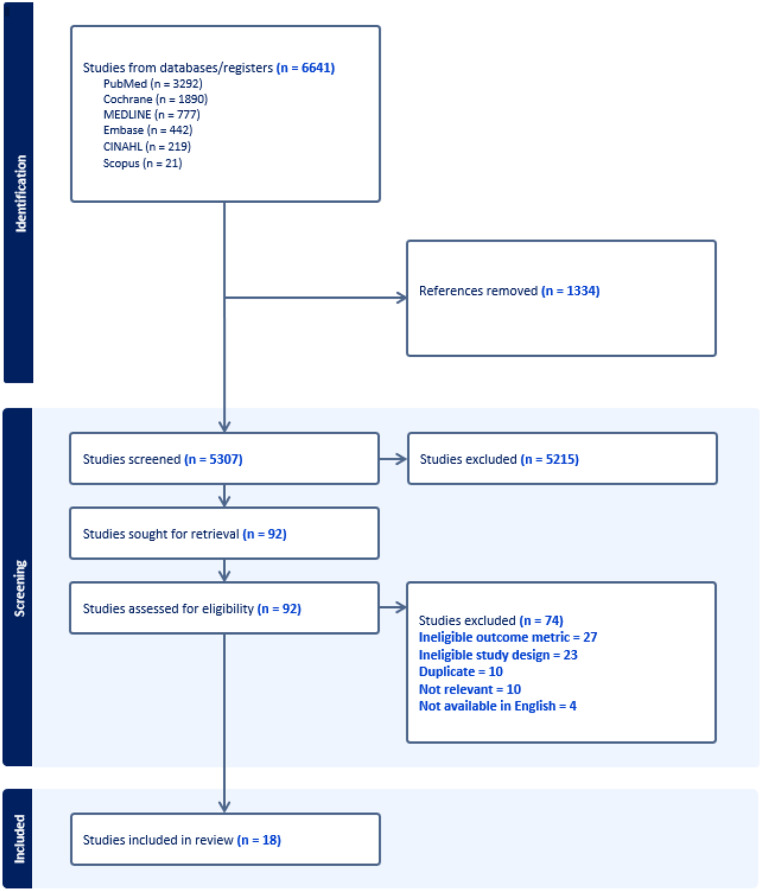
PRIMSA Flow Diagram outlining the identification and screening process for included studies

Adherence to the Preferred Reporting Items for Systematic Review and Meta-Analysis (PRISMA) Statement guided the reportage process for this systematic review and meta-analysis. After the removal of duplicate entries, the resultant articles were independently scrutinized for suitability by two independent authors using Covidence systematic review software. Initial screening involved assessing article abstracts to determine their relevance to the study criteria. If an abstract did not provide enough information for a conclusive decision, a full-text review was undertaken to ascertain its eligibility. After independent screening, the two authors compared their findings. Any discrepancies in the selection of studies were resolved through discussion or, if necessary, by consulting a third, impartial investigator. This rigorous process was implemented to minimize bias and ensure the validity of study selection. The following information was recorded: authors, year of publication, country, study design, sample size, characteristics of participants (age, gender), educational modalities used, outcome measures, and key findings. The search diagram is summarized in Fig. 1.

### Primary outcome

The primary outcome of our meta-analysis was the effect of various educational modalities on atopic dermatitis severity. This outcome was evaluated using multiple scales across the studies, among the most common including SCORAD, Dermatology Life Quality Index (DLQI), Patient Oriented Eczema Measure (POEM), and Eczema Area and Severity Index (EASI).

SCORAD is a well-regarded tool for quantifying patients’ the severity of disease by categorizing a point system respective to extent of lesions, itch, and sleep quality of life among others. The DLQI scoring scale is a commonly used measure to assess the extent of disease severity among different skin conditions with categories focused on disease severity and patient quality of life. The POEM scale is another AD-specific severity scale with a focus on subjective patient and/or caregiver reported measures.

Given the variety of scales used in the included studies, a direct, uniform comparison of disease intensity was not possible. However, each scale contributes unique insights into the experience and severity of atopic dermatitis severity. This rich array of measurements allowed for a more comprehensive understanding of disease intensity and the effectiveness of various interventions.

The results from each study were converted to a common effect size metric to facilitate comparison. Each scale’s relative strengths and possible limitations were considered during data interpretation. As such, our primary outcome analysis not only encompasses a broad overview of the treatment effectiveness but also captures the multidimensional nature of itch as experienced by patients.

### Quality assessment

The quality of included studies was evaluated using the **Cochrane Risk of Bias Assessment tool.** This tool evaluates the risk of bias in clinical studies based on various domains. Any disagreements between the two authors in the quality assessment were resolved by discussion or by involving a third author.

### Statistical analysis

Statistical analyses were performed using Review Manager 5.4.1 (Cochrane Collaboration). Heterogeneity among studies was quantified using the I^2^ statistic, τ^2^ and the χ^2^ statistic. Studies were regarded as homogenous if I^2^ ≤ 40%, in which case a fixed effect model of meta-analysis was utilized. If I^2^ > 40%, a random effect model was used. Subgroup analysis was conducted based on the different treatment modalities. Sensitivity analysis was performed by sequentially omitting each study to assess the robustness of the results. Publication bias was evaluated visually with a funnel plot and statistically with **Egger’s regression test.**

## Results

The present systematic review and meta-analysis synthesizes findings from a total of eighteen studies, representing a comprehensive pool of 3,234 study subjects, with 1,597 in the active arm. From our research, this is the first meta-analysis investigating the efficacy of online educational interventions for the management of atopic dermatitis as compared to traditional in-person interventions.

### Study characteristics

The included studies encompassed a wide range of educational interventions for atopic dermatitis, providing a diverse and multifaceted data pool. The methodologies, population demographics, and AD outcome scales varied among studies, offering a nuanced and thorough investigation of the phenomenon that can be seen in Table 1. Most studies were randomized controlled trials, primarily from North America and Western Europe. Most interventions were focused on patient and/or caregiver education about disease management, self-care techniques, avoidance of triggers, and comprehensive understanding of the disease process. Some studies also incorporated psychological support or group discussions to foster emotional well-being and collective problem-solving. The age of study participants ranged from infancy to adulthood, with a substantial portion of studies focusing on pediatric populations (mean age 7.15; range 2.40-34.53).

**Table 1:** Study characteristics

Author, Year	Mean Age (years)	Treatment Modality Used	Length	Outcome Measurement Scale
Staab, 2002 [3]	2.9	Parental Training Program	6 Sessions; 2 h. each	SCORAD^a^, Treatment Behavior Questionaire
Grillo, 2006 [4]	4.3	Parental and Patient Training Course (Combined)	1 session; 2 h.	SCORAD, DFI^b^, CDQOL^c^, IDQOL^d^
Staab, 2006 [5]	2.4; 14.9*	Parental and Patient Training Course (Separate + Combined)	6 Sessions; 2 h. each	SCORAD, Subjective Severity, Itch Behavior, Parental QoL^e^
Weber, 2008 [6]	6.61	Parental and Patient Training Courses (Separate and then Combined)	Biweekly for 6 months	CDLQI, FDI^f^, Pruritus Questionnaire
Shaw, 2008 [7]	5.48	Parental and Patient Counseling + Support Line	15-minute session; optional 24/7 support line for 90 months	SCORAD, CDLQI, IDQLI
Moore, 2009 [8]	8.25	Parental and Patient Eczema Workshop (Nurse-led)	1 session; 90-minute avg.	SCORAD
Armstrong, 2011 [9]	48	Online Video Education	Access anytime over 12 weeks	POEM^g^
Futamura, 2013 [10]	2.4	Parental Education	5 sessions over 2 days; 260 min total	SCORAD, DFI
Armstrong, 2015 [11]	27.7	Online Medical and Educational Model	12-month period	POEM, IGA^h^
Pustišek, 2016 [12]	1.39	Parental Education	1 session; 2 h.	SCORAD, PSS^i^, STAI^j^, FDLQI^k^
Gilliam, 2016 [13]	4	Eczema Action Plan	N/A	Child Eczema Study Questionnaire
Heratizadeh, 2017 [14]	34.53	Adult Educational Programming	12 h.	SCORAD, DLQI
Liang, 2018 [15]	5.6	Parental Educational Intervention	4 sessions; 2 h.	SCORAD, IDLQI, CDLQI
Cheng, 2020 [16]	5.81	Parental Educational Booklet + Online Platform Education (Nurse led)	20 min for booklet teaching; 10-minute video; 3 months of online forums	SCORAD, C-FDLQI, C-PASECI
Muzzolon, 2021 [17]	4.75	Parental and Patient Educational Program (Combined)	1 session; 90 min	SCORAD, EASI, IDQOL, CDLQI, DFIQ
Lebovidge, 2021 [18]	3.7	Caregiver Handbook	N/A	PASECI^l^, POEM, EASI, IDQOL/CDLQI
Santer, 2022 [19]	6.8; 19.3*	Virtual Eczema Education	N/A	POEM
Chen, 2023 [20]	3	Community Health Worker	2 sessions in-person; 60–120 min; follow up telephone calls; 5 min	SCORAD, Sp-PIQoL-AD^m^
Singer, 2018 [21]	0.9	Parental Text Messaging	42 daily texts or until follow-up	EASI, AD Knowledge Quiz
Hedman-Lagerlöf, 2021 [22]	37	Patient Online CBT Modules	12 week access	POEM, DLQI

^a^SCORAD – SCORing Atopic Dermatitis, ^b^DFI – Dermatology Family Impact Questionnaire, ^c^CDQOL – Children’s Dermatology Life Quality Index, ^d^IDQOL – Infants’ Dermatitis Quality of Life Index, ^e^QoL – Quality of Life, ^f^FDI – Parental Functional Disability Inventory, ^g^POEM – Patient Oriented Eczema Measure, ^h^IGA – Investigator Global Scale, ^i^PSS – Perceived Stress Scale,^j^STAI – State-Trait Anxiety Inventory, ^k^FDLQI – Family Dermatology Life Quality Index, ^l^PASECI – Parental Self-Efficacy with Eczema Care Index, ^m^Sp-PIQoL-AD – Spanish-language Parental Quality of Life Questionnaire for Atopic Dermatitis, *Adult Arms

### Outcome assessment

Outcome assessment was predominantly conducted using the SCORAD index, although several studies employed alternative or additional measures such as the EASI, POEM, and DLQI.

#### SCORAD index

**Fig. 2:**
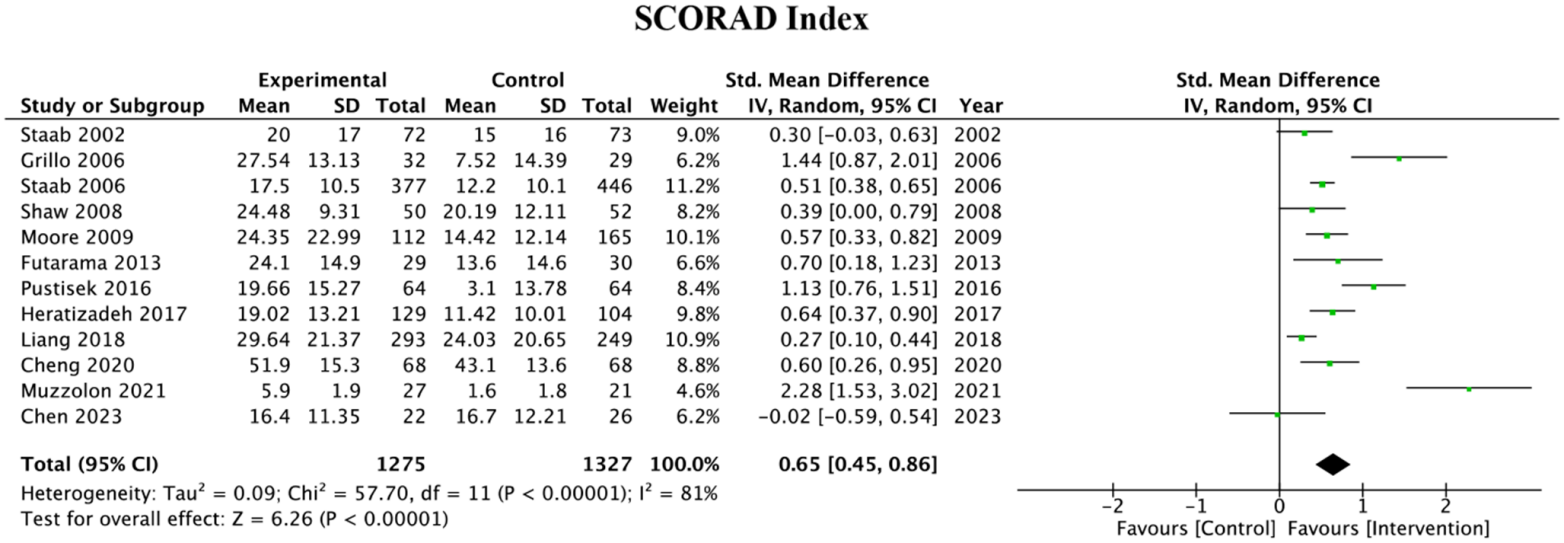
SCORAD primary outcome measures of 12 studies and associated forest plot

Twelve of the studies used the SCORAD index as their primary outcome measure. A summary of the effects of the various educational programs on the SCORAD index across these studies indicated a significant reduction in the SCORAD index in the intervention group compared to the control group (standard mean difference [SMD] = 0.65, 95% Confidence Intervals (CI): 0.45–0.86), P < 0.00001). High heterogeneity was observed among the studies (I^2^ = 81%). The pooled effect can be observed in Fig. 2.

#### QOL

**Fig. 3:**
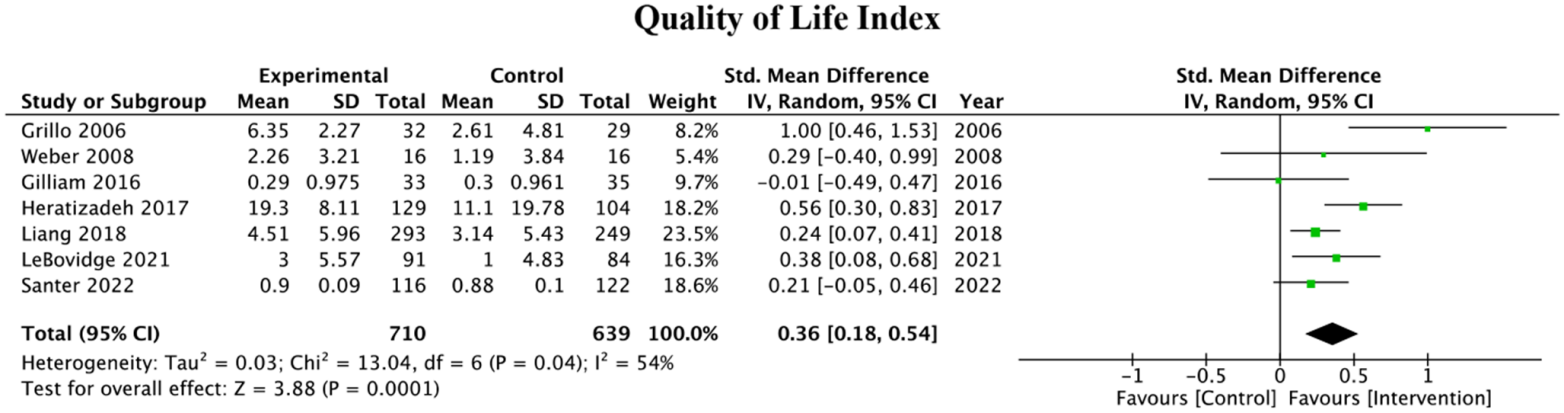
Quality of Life outcome measures of 7 studies and associated forest plot

Among the studies assessed, seven assessed the quality of life as an outcome measure, using various indices (CDLQI, FDLQI, and IDQOL, DLQI, CHU-9D, Skindex-29). The meta-analysis of these studies revealed a significant pooled SMD of 0.36 (95% CI: 0.18–0.54, P = 0.0002), with high heterogeneity between the studies (I^2^ = 54%). The pooled effect can be observed in Fig. 3.

#### POEM

**Fig. 4:**
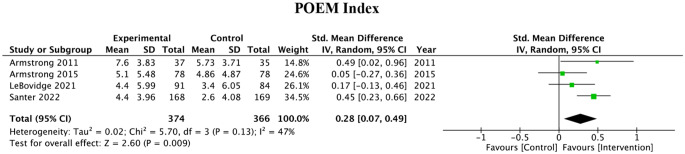
POEM outcome measures of 4 studies and associated forest plot

The POEM score was reported as outcome measure in four studies. These studies reported a significant reduction in the POEM score in the intervention group (SMD = 0.28, 95% CI: 0.07–0.49, P = 0.009). Moderate heterogeneity was observed between these studies these (I^2^ = 47%). The pooled effect can be observed in Fig. 4.

#### EASI

**Fig. 5:**
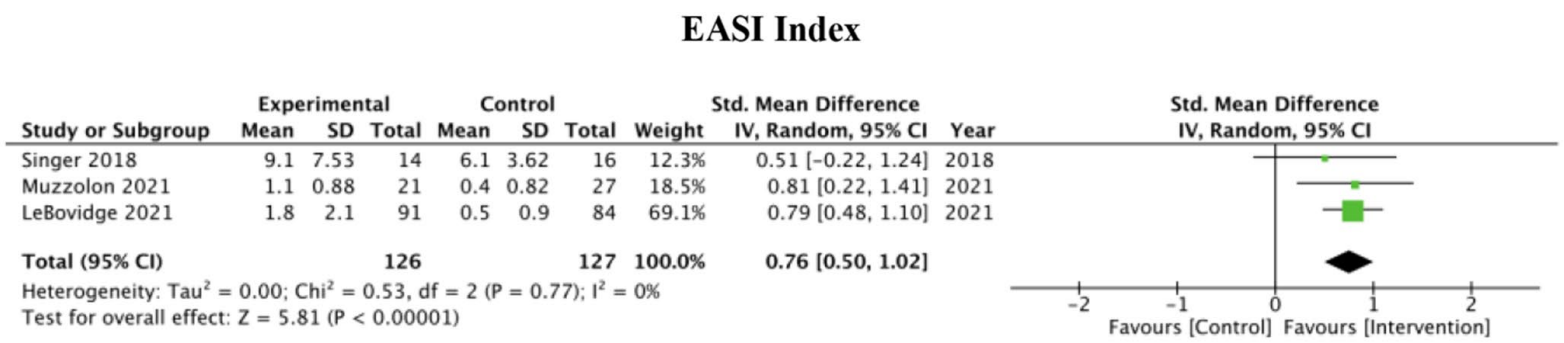
EASI outcome measures of 3 studies and associated forest plot

EASI measurements were employed in only three studies. All three studies reported a significant reduction in EASI scores in the intervention group compared to the control group. The pooled mean difference was 0.80 (95% CI: 0.54–1.06, P < 0.00001), with low heterogeneity between the studies (I^2^ = 0%). The pooled effect can be observed in Fig. 5.

### Subgroup analyses

**Fig. 6:**
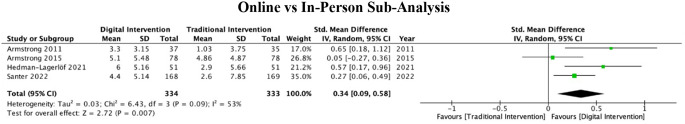
Sub-analysis of studies based on online vs. in-person intervention utilizing the POEM index

Subgroup analyses were conducted based on the mode of educational intervention, contrasting virtual and in-person methods. Due to the limited number of studies using virtual methods, the POEM score was utilized for this comparison as it was the most reported measure in these studies. The results demonstrated a beneficial effect of both in-person and virtual educational programs on atopic dermatitis management. However, the reduction in POEM scores in the virtual intervention group was not statistically significant as shown in Fig. 6.

**Fig. 7:**
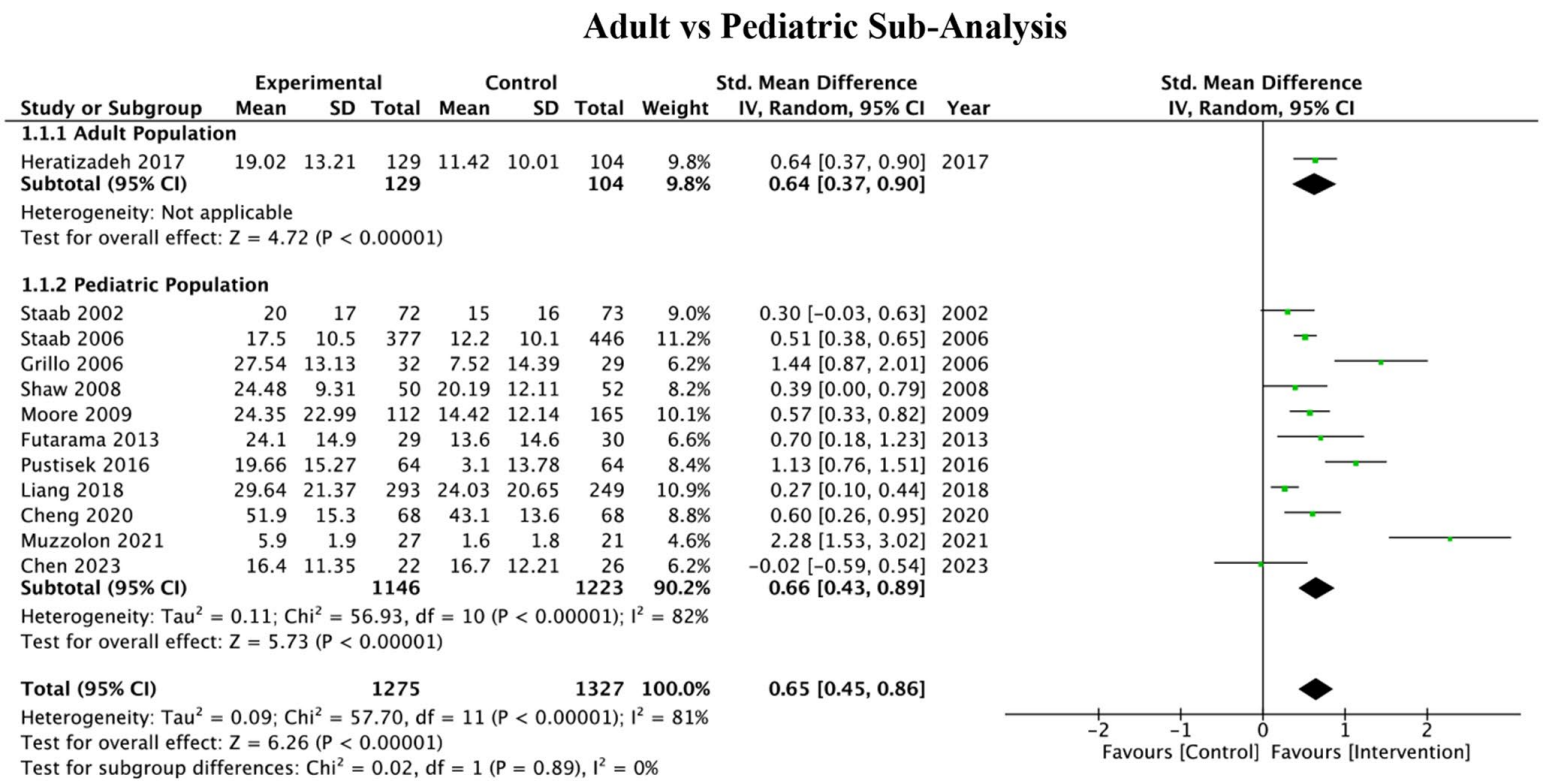
Sub-analysis of studies differentiating between adult vs. pediatric populations using the SCORAD index

When performing subgroup analyses based on the age of the patient populations, groups were differentiated between adult and pediatric populations. Both demographics showed beneficial reductions in the SCORAD index following the educational interventions. However, the applicability of these findings to the adult population is limited, as only one study focused on this demographic (Fig. 7).

### Heterogeneity and sensitivity analysis

Our analysis showed varying levels of heterogeneity among the different outcome measures. The high level of heterogeneity in the SCORAD index and DLQI score could be attributed to several factors including study design, measurement protocols, sample size, participant characteristics, duration of follow-up, and type of educational interventions. In contrast, the POEM score and EASI measurements showed lower levels of heterogeneity suggesting a higher level of consistency among these studies.

To evaluate the robustness of our results, a sensitivity analysis was conducted. Each study was excluded one at a time to evaluate its influence on the overall effect size. The overall effect size remained consistent and did not show significant variation, thereby attesting to the stability of the findings.

### Publication bias

**Fig. 8:**
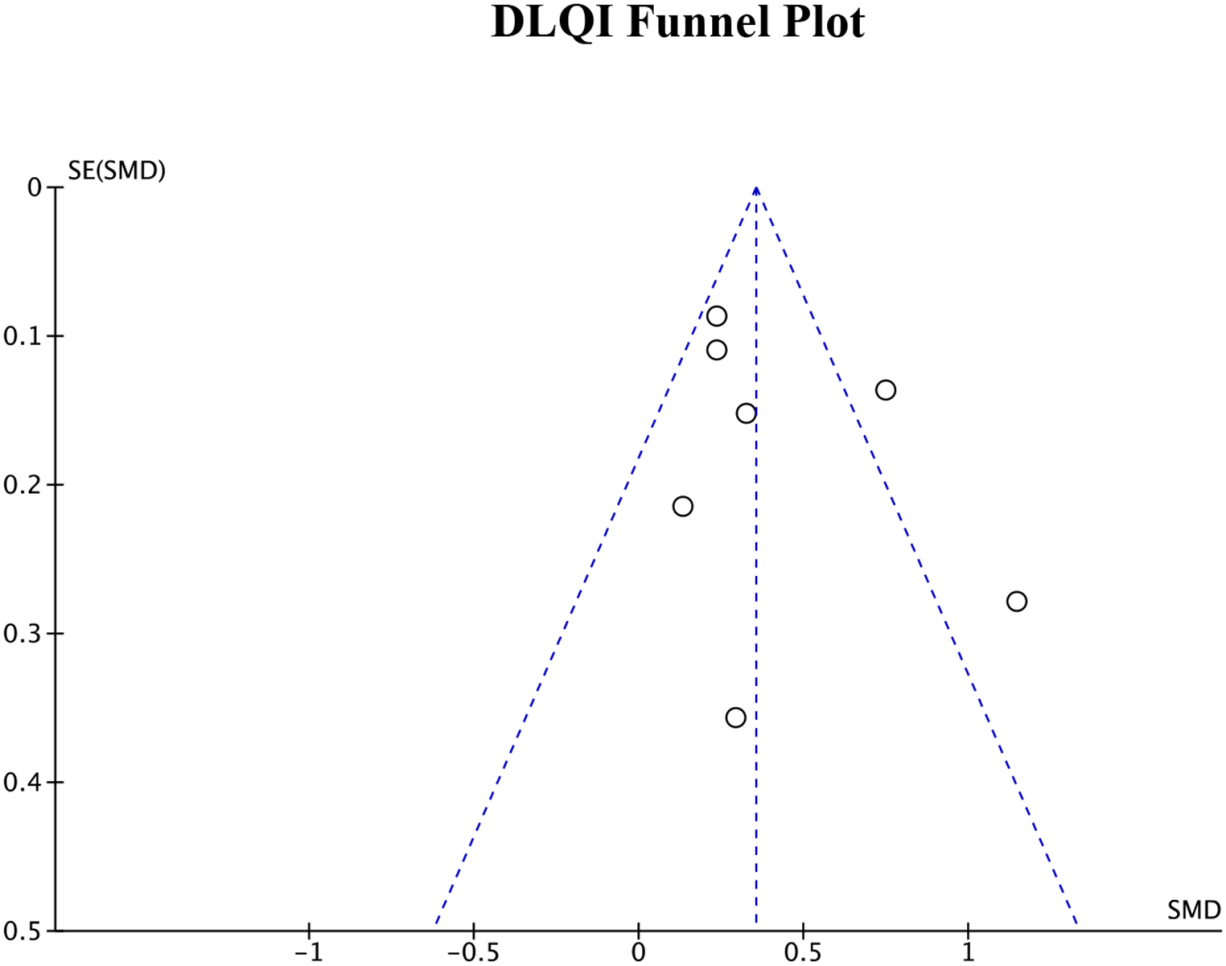
Funnel plot for DLQI score representing visual analysis of any publication bias

**Fig. 9:**
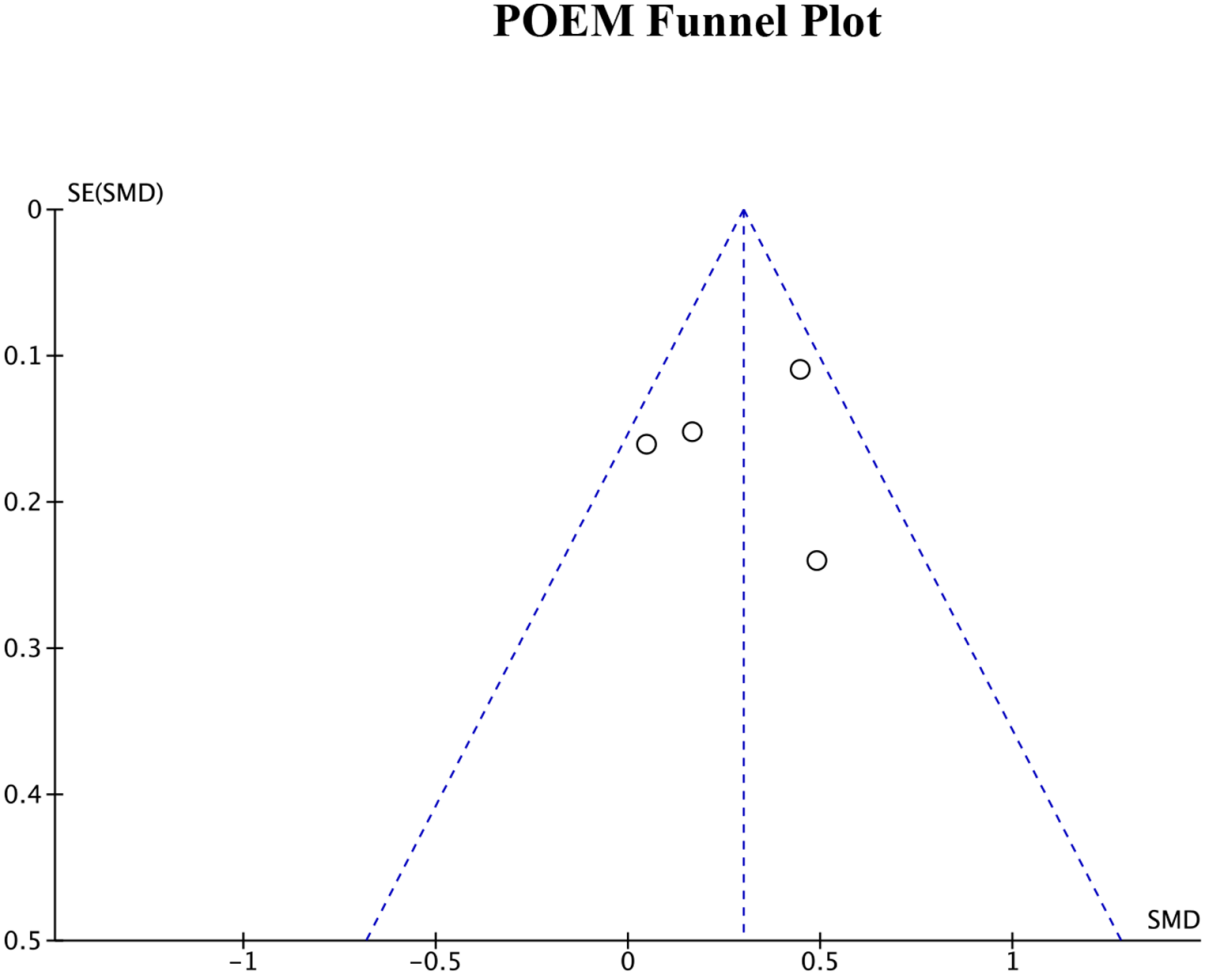
Funnel plot for POEM score representing visual analysis of any publication bias

**Fig. 10:**
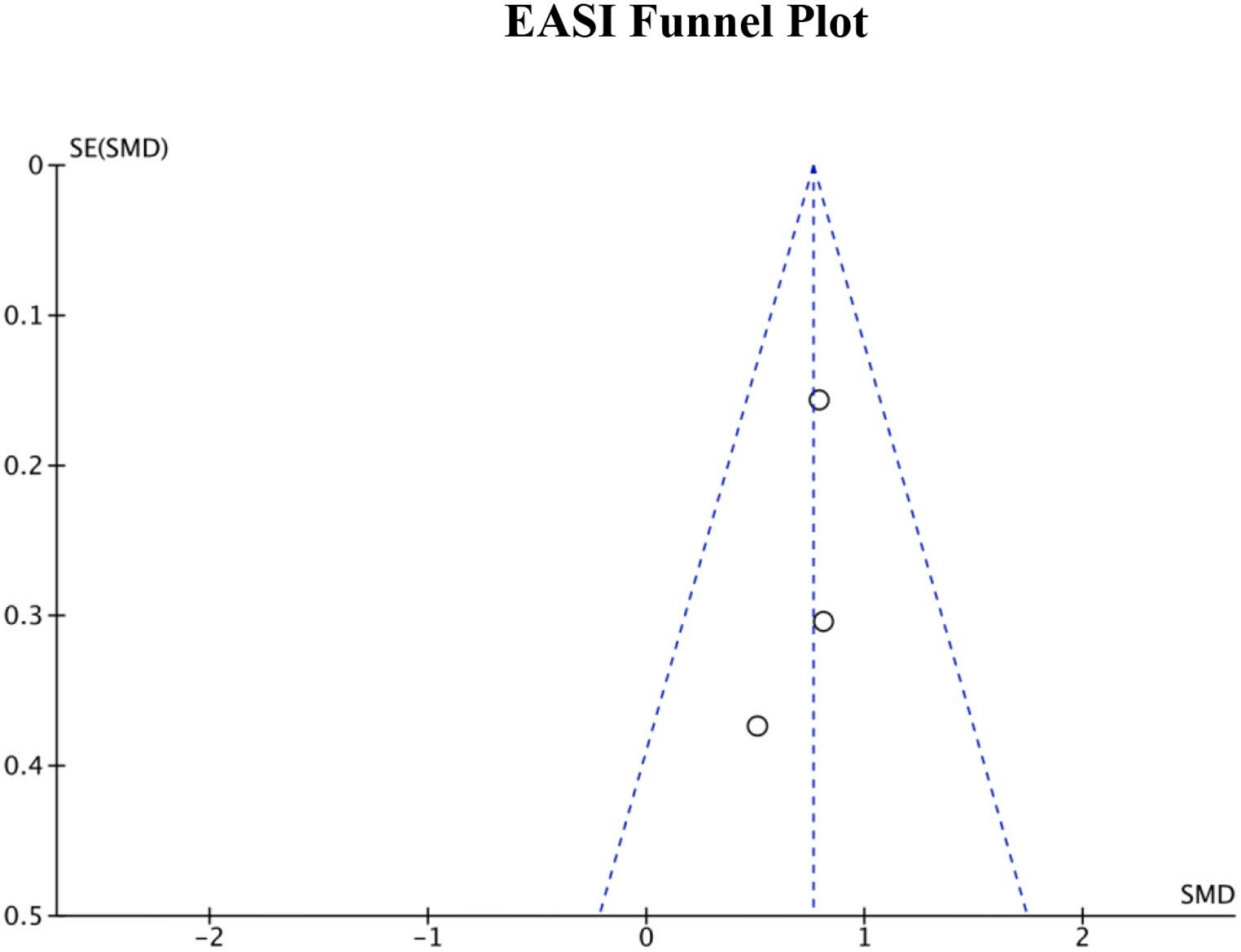
Funnel plot for EASI measurements representing visual analysis of any publication bias

**Fig. 11:**
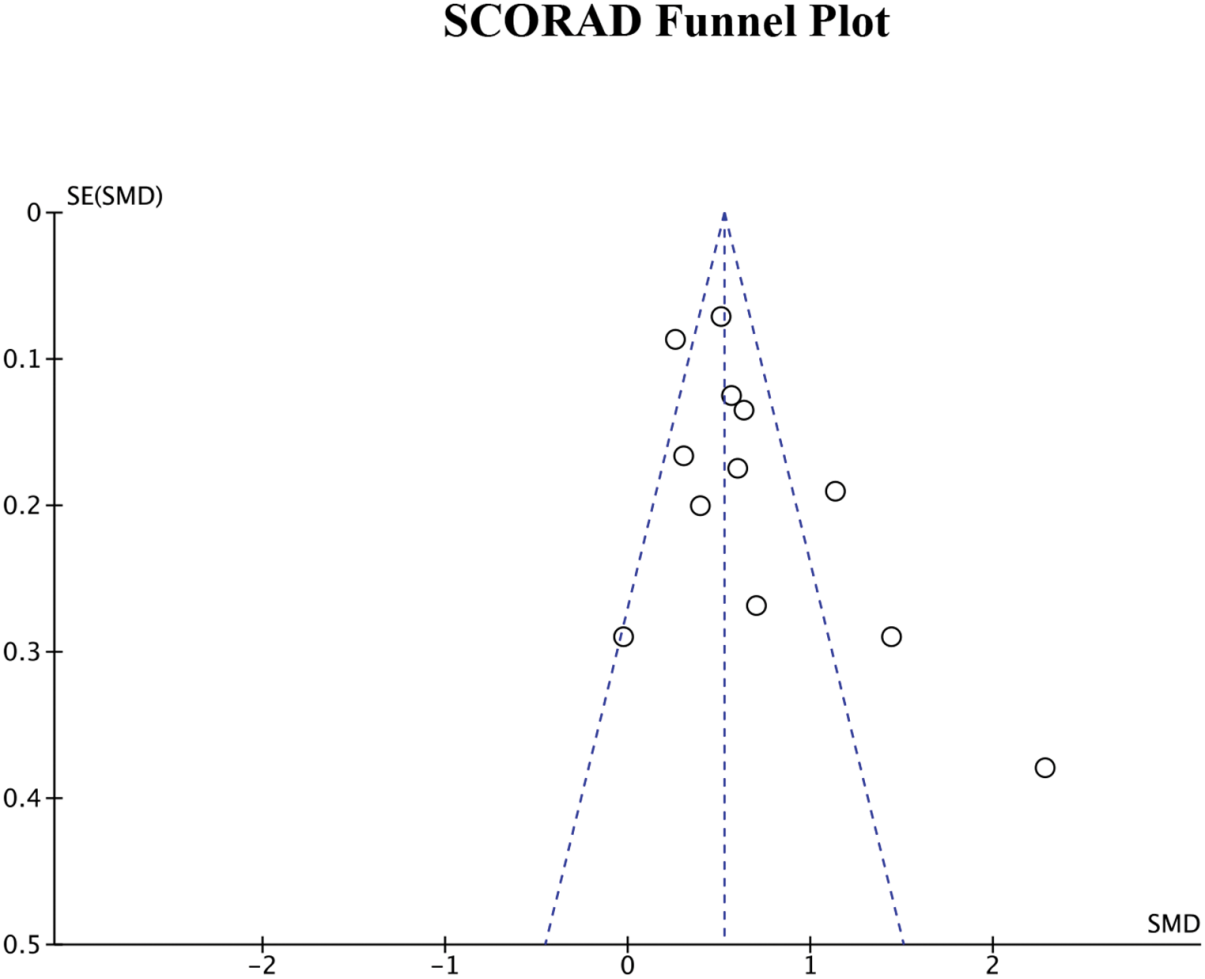
Funnel plot for SCORAD index representing visual analysis of any publication bias

Publication bias was assessed by generating funnel plots for each of the four primary outcome measures. The funnel plots for DLQI score, POEM score, EASI measurements, and SCORAD index can be seen in Figs. 8, 9 and 10, and Fig. 11, respectively. Visual inspection of these funnel plots did not suggest any clear asymmetry, indicating the absence of notable publication bias for these outcomes. Moreover, the Egger’s regression test provided statistical evidence supporting this visual interpretation. The p-values for the Egger’s test for the SCORAD index, DLQI score, POEM score, and EASI measurements were 0.0677, 0.2718, 0.4676, and 0.312, respectively, suggesting a lack of significant publication bias for these outcomes. While our analysis does not detect substantial publication bias, due to the small number of studies, it cannot definitively rule it out. Further research and more comprehensive data collection are necessary to conclusively address the potential issue of publication bias in this field.

## Discussion

This meta-analysis collected studies from current literature to determine the effectiveness of educational interventions regarding the severity of AD and quality of life. Compared to a prior meta-analysis performed in 2020, our study had no restriction on age, included interventions that featured adult participants, and performed a sub-analysis based on virtual modalities [2]. Additionally, our study included a larger study size in the quantitative analysis with up to 18 studies total compared to the 7 studies analyzed for SCORAD and 4 for quality of life in the past review.

Table 1; Fig. 2 showcase a summary of the 12 articles which used SCORAD as a metric for the impact of the intervention. Of these 12 articles, 11 articles showed a significant improvement from baseline with AD education intervention.

Muzzolon et al. examined the use of an educational workshop for pediatric patients and parents in improving the quality of life and disease severity for pediatric patients with AD. Of all the studies which used the SCORAD scoring method, this study had the largest improvement in scores. The 90-minute educational intervention consisted of two stages: one with patients accompanied by parents and another with just patients. During the meeting with the parents, they attended a lecture discussing the general aspects of AD, including pathophysiology, triggers, and treatment, followed by an open discussion regarding their personal experiences with AD. At the pediatric patients’ meeting, they participated in expressive activities such as drawing and painting about their experience living with AD. Later, they watched a puppet show which focused on the importance of care practices for managing AD and reproduced the puppet show through their drawings. Finally, they collaboratively participated in a hydration workshop on proper application of moisturizers with the hopes of increasing education and mutual support amongst the group. While most studies solely focus on parent education of AD, this study highlights the importance of providing education to both the patient and caregiver by incorporating unique educational resources relevant to each stakeholder.

Chen et al. studied the impact of educational follow-up with community health workers over a 12-week period in addition to standard clinic patient education for Hispanic patients living with AD. Of all the studies which used the SCORAD scoring method, this study had the least significant results with no difference between the control and active arm. One possible limitation in the protocol included a lack of standardization of educational protocol between the educators which may have played a role in the active arm not producing significant improvements over the control. Due to increased health disparities in Hispanic patients living with AD [21, 22], culturally competent public health interventions are vital. Prior studies in the AD educational realm have shown success when implementing cultural phenomena as part of a tailored intervention [23]. Future studies using models like community health workers should be encouraged in combination with standardized educational programming and additional forms of active learning.

Moreover, there was a large degree of heterogeneity (I^2^ = 81%) among the studies which used SCORAD as a scoring system for the impact of AD education on AD severity. This large degree of heterogeneity can be attributed to the various patient populations that were sampled from the different studies (pediatric patients, adult patients, and parents of pediatric patients).

Altogether, the standardized mean difference of 0.65 between all the pooled SCORAD studies suggests that educational interventions have a ‘moderate’ statistical improvement regarding changes in this specific outcome metric. In particular, the overall mean improvement was Z = 6.26 (p < 0.00001), which at first glance falls short of what is considered a minimally important change (MIC) for absolute improvements of SCORAD (8.2) [24]. An important factor to consider, however, is that the best criteria for MIC can vary based on disease severity. For example, patients with mild disease severity of score 8 or less cannot reach a statistical improvement greater than 8 regardless of reported clinical improvement [25].

Table 1; Fig. 3 summarize 7 studies that used Dermatology Life Quality Index (DLQI/QoL) as a metric for intervention effectiveness. Of the 7 articles, 5 showed statistically significant improvements in the DLQI/QoL index with the use of educational interventions. Similarly, the Patient-Oriented Eczema Measurement (POEM) is a questionnaire used to assess the severity and impact of atopic dermatitis on the daily life of patients. Table 1; Fig. 4 examine the 4 analyzed studies using POEM as a measure to determine the impact of educational interventions on AD severity. 2 of these 4 articles showed significant improvements in scores with the use of educational interventions.

In another study, Grillo et al. examined the use of a 2-hour educational workshop on the appropriate care for AD. The workshop was a practical session focusing on wet wrapping and cream application. Of the 7 studies which measured DLQI/QoL and POEM, this study had the greatest improvement. Quality of life measures were significantly improved only in the group aged 5–16 years. Furthermore, Gilliam et al. studied the use of Eczema Action Plans (EAPs) to improve parental/caregiver education on treatment and care for children with AD. This study showed the least improvement in AD severity for interventions measuring DLQI/QoL and POEM with no significant difference between the active and control arms. While EAPs and patient pamphlets might be a simple method to help patients understand eczema and adhere to medication regimens, the trend observed over the various scoring metrics illustrate that active methods of education are superior to passive learning gained through a pamphlet format, regardless of whether the pamphlet content is distributed through in-person or virtual means.

Figure 5; Table 1 display the 3 studies that used the EASI scoring system to measure the impact of atopic dermatitis educational interventions on disease severity. Notably, Singer et al. showed how the effect of daily text messages with patient education material and treatment reminders plays a significant role in reducing AD severity, as measured through EASI. Of the 3 studies we examined, this study showed the largest standard mean difference. However, the authors concluded that this pilot study did not demonstrate a significant difference in EASI scores for text message reminders as reflected by the low standard mean difference. Comparably, the study with the lowest mean in our EASI analysis had a similar overall mean. LeBovidge et al. randomized 175 caregivers into an intervention group (handbook in addition to standard AD management) and a control group (standard AD management). They did not demonstrate a great difference in EASI with a standardized mean difference of 0.79 and a 95% confidence interval of 0.48–1.10. One possible explanation for lower scores among the EASI groups could be low sample size in combination with passive forms of education (e.g. text messaging, handbooks) constituting most of the interventions within this cohort.

Most educational interventions in the literature have focused on the pediatric population, and while the pediatric population has the highest prevalence of AD, adult patients who have this diagnosis typically experience more complex forms of the disease [26–28]. Although we were unable to perform a high-powered analysis from lack of adult interventions utilizing standardized metrics compared to pediatric cohorts, our sub-analysis provided in Fig. 7 shows that there was an equal improvement in adult educational interventions when compared to pediatric populations for SCORAD outcomes. These findings suggest that targeted educational programs in understudied adult populations can be just as effective as pediatric-focused ones and should be explored further.

With the advent of the COVID-19 pandemic, the utilization of online formatting has put telehealth at the forefront. Similar interventions on a patient education level can help patients access programming that might otherwise be inaccessible due to distance, availability, or financial costs associated with attending (e.g. eczema schools located in limited urban settings). Our sub-analysis comparing in-person vs. virtual education arms outlined in Fig. 6 found no significant differences between groups. These results suggest that virtual interventions can be effective vehicles for content delivery and reducing barriers of access to patient education. More investigations using virtual interventions should be conducted to allow a higher-powered analysis.

Overall, educational interventions were shown to have significant impacts on reducing AD severity as per the SCORAD, DLQI/QoL, POEM, and EASI measurements. The studies which focused on interventions with multiple learning modalities including practical skills and collaborative learning had the greatest effect. Future studies on the impact of educational interventions should aim to incorporate various methodologies to better stimulate individual learning preferences. Research in adult cohorts and the effectiveness of virtual interventions should be further investigated.

## Data Availability

No datasets were generated or analysed during the current study.
